# The incidence of thrombosis with co-occurring thrombocytopenia prior to the SARS-CoV2 pandemic: A population-based study

**DOI:** 10.1371/journal.pone.0301359

**Published:** 2024-05-24

**Authors:** Yanfang Liu, Choo-Hua Goh, Dereck Shen, Hong Qiu, Kuan-Chih Huang, Man Luo, Zhangjing Chen, Chao-Hsiun Tang

**Affiliations:** 1 Department of Global Real-World Evidence, Janssen Pharmaceuticals LLC, Raritan, New Jersey, United States of America; 2 Global Epidemiology, Office of the Chief Medical Officer, Johnson & Johnson, Singapore, Taiwan; 3 School of Health Care Administration, College of Management, Taipei Medical University, Taipei, Taiwan; 4 Janssen China Research & Development, Shanghai, China; University of Illinois College of Medicine at Peoria, UNITED STATES

## Abstract

**Background:**

Thrombosis with thrombocytopenia syndrome (TTS) is a very rare prothrombotic disorder that is a safety concern for some COVID-19 vaccines. We aimed to devise a case definition to estimate the incidence of thrombosis with thrombocytopenia as a proxy for TTS in a national insurance claims database.

**Methods:**

We conducted a retrospective observational study using the National Health Insurance Research Database (NHIRD) in Taiwan over the three-year period prior to the SARS-COV-2 pandemic (2017–2019). Our case definition was all patients with newly diagnosed thrombosis co-occurring with a diagnosis of thrombocytopenia within seven days before or after the thrombosis diagnosis. Cases were identified using International Classification of Disease-10 codes.

**Findings:**

We identified 2010 patients with newly diagnosed thrombosis co-occurring with thrombocytopenia during the study period. The mean age was 64.71 years; female:male ratio 1:1.45. The most frequent thrombotic events were coronary artery disease (18.81%), cerebral infarction (16.87%), and disseminated intravascular coagulation (13.13%). Cerebral venous sinus thrombosis was rare (<0.1%). The average annual incidence rate of co-occurring new diagnoses of thrombosis and thrombocytopenia was 2.84 per 100 000 population. Incidence rates were higher in men than women, except in 20–39 year-olds (higher in females). 20.6% of patients died within the first month after diagnosis.

**Interpretation:**

We observed that the demographic and clinical characteristics of thrombosis with co-occurring thrombocytopenia using our case definition is different from that of TTS. Further research is needed to refine the case definition of TTS in the post-COVID-19 vaccination period.

## Introduction

The evaluation of vaccine safety post-authorisation is essential to identify rare and very rare adverse events that can only be detectable once the vaccine has been used in large and diverse populations over a longer period of time. Healthcare claims databases are a potentially rich source of safety information because they capture healthcare records for very large populations [1]. Additionally, the collection of data is longitudinal, allowing follow up of outcomes before and after an exposure and the estimation of rates of incident events. Many potential confounders, including age, sex, co-morbidities and medication use can be also accounted for, and analyses can be conducted rapidly, repeatedly, and at a lower cost than targeted prospective safety studies with primary data collection. Potential limitations of research using healthcare databases are that reimbursement is based on International Classification of Disease (ICD) codes with the assumption that diseases and their complications are correctly coded, and that patients with the same code have similar disease, as well as similar investigative and treatment requirements. Clinical information including imaging and laboratory test results and clinical reasoning are lacking in claims databases, which potentially limits clinical application of the findings.

Thrombosis with thrombocytopenia syndrome (TTS) is a newly described and very rare entity that has been observed in recipients of some coronavirus disease 2019 (COVID-19) vaccines [2]. Thrombotic events in TTS frequently occur in uncommon sites, such as the cerebral venous sinuses, splanchnic, deep, and internal jugular veins [3]. Thrombosis occurs concurrently with thrombocytopenia in the presence of antibodies to platelet factor 4 (PF4). Women are affected more than men, and younger persons more than older persons [3]. Mortality is high, reported to be 39% after TTS with cerebral venous thrombosis [4].

Post-marketing surveillance of TTS presents unique challenges. The rarity of the condition means that large populations are required to study TTS and prospective post-marketing safety studies with primary data collection are not feasible. Healthcare claims databases that hold information on large populations are potential candidates for monitoring TTS rates, but the syndrome was not recognised prior to COVID-19 vaccination implementation and no specific ICD-code exists. Due to its extreme rarity and diverse clinical presentation, characterising the clinical syndrome of TTS has proven challenging [2,3]. There have been several attempts to identify case definitions by, for example, the Brighton Collaboration, the British Society of Haematology, the American Society of Hematology and United States Centers for Disease Control and Prevention (CDC) [5–8]. The Level 1 (highest level of certainty) Brighton TTS interim case definition is based on laboratory evidence of thrombocytopenia <150 x10^9^/L with no known recent exposure to heparin, with radiologically-confirmed thrombosis/thromboembolism, *or* persistence headache with elevated D-dimer, *or both* a clinical presentation consistent with site-specific symptoms of thrombosis and raised D-dimer *or* positive anti-PF4 [5]. Similar definitions are used by the British and American Societies, whereas the CDC defines cases according to the anatomical location of the thrombosis, assigning Tier 1 to unusual sites such as cerebral venous sinuses, portal vein and splenic vein and Tier 2 for other locations. All definitions require evidence of thrombocytopenia and anti-PF4 antibodies [5–8]. Clinical symptoms, laboratory and radiology results that contribute to case definition criteria–including measures of platelets and anti-PF4 antibodies–are not captured in health claims and previous algorithms to identify TTS in claims data have not been validated against medical records. We developed an algorithm to estimate the incidence of thrombosis with co-occurring thrombocytopenia as a proxy for TTS as a possible monitoring tool in the post-vaccination era. Given the rarity of TTS, we sought to create a sensitive definition to maximise detection of possible cases. We defined thrombosis with co-occurring thrombocytopenia as newly diagnosed thrombosis co-occurring with a diagnosis of thrombocytopenia within seven days before or after the thrombosis diagnosis. We applied the definition in Taiwan’s National Health Insurance Research database (NHIRD), a high-quality, population-based healthcare claims database that covers >99% of the population of Taiwan [9]. The aim of the study was to assess the utility of the definition as a proxy for TTS by describing the incidence rate of co-occurring thrombosis with thrombocytopenia.

## Methods

### Study population and design

Taiwan’s National Health Insurance (NHI) programme is a single-payer system. Administrative and claims data are held centrally in the NHIRD provided by the Taiwan National Health Insurance Administration, and maintained by the Health and Welfare Data Science Center, Ministry of Health and Welfare, Executive Yuan, Taiwan [10]. The NHIRD is linked at the patient level to Taiwan’s Death Registry.

Our definition of diagnosis of thrombosis with a co-occurring diagnosis of thrombocytopenia was defined as a patient who experienced a newly diagnosed thrombotic event (outpatient, inpatient or emergency room settings) with newly diagnosed thrombocytopenia that co-occurred within a 7-day period before or after the thrombosis diagnosis date (the index date). The following steps were applied: First, new thrombotic events were identified in the NHIRD by a single inpatient or outpatient claim with a primary or secondary diagnosis using one of the following ICD-10 codes: G43.6, D65, D73.5, E88.41, F01.1, G08, G43.6, G45, G46, H31.3, H34, I20-I26, I60-I66, I67.6, I67.82, I69.3, I70.92, I74, I75, I80-I82, I87, K55, K76.5, M31.1, M47.0x, N28.0, N48.81, O03.85, O08.7, P60, P91.0, Q27.1 that occurred between 01 January 2017 until 31 December 2019 (the first case of COVID-19 disease in Taiwan was identified on 21 January 2020). Second, new thrombocytopenia events were identified by a single inpatient or outpatient claim with a primary or secondary diagnosis using one of the following ICD-10 codes D69.3, D69.4, D69.5, D69.6, or by any outpatient or inpatient claim with platelet replacement therapy (NHIRD reimbursement code: 93004C), regardless of the diagnosis codes in the claim. Diagnoses were considered ‘new’ from 01 January 2017 until 31 December 2019 if there were no prior events of any thrombosis or of thrombocytopenia due to any cause (D60, D61, D69.3, D69.4, D69.5, D69.6, D75.82, D82.0, O14.2 or 93004C) in the 12 months prior to the index date. Because of the frequency of coronary artery occlusion, events with code I20, I24 and I25 were considered new if there were no prior claims for these codes from January 2016, coinciding with the adoption of ICD-10 coding in the NHIRD.

Patients were excluded if they had a history of prior thrombocytopenia from any cause, if they did not have evidence of thrombocytopenia, or if they had thrombocytopenia but not within seven days before or after the index date.

A unique and anonymous identifier was used to link each patient in the NHIRD with the Death Registry. All deaths in Taiwan are legally required to be registered according to ICD-10 codes within 4 weeks after the patient’s death. All patients were followed until death, withdrawal from the NHIRD, or until the end of the study (31 December 2019), whichever occurred first.

Demographic characteristics and the presence of comorbidities were assessed during a 12-month baseline period before the index date. Comorbidities were identified using ICD-10 codes with at least two outpatient visits and/or one inpatient hospitalization record, and/or one emergency room visit. Pregnancy status was ascertained from records of prenatal outpatient visits 6 months prior to the index date.

The study was approved by the Taipei Medical University Joint Institutional Review Board (TMU-JIRB No. N202109054, 10 November 2021). This study was granted an exemption from the need for patient consent and from ethical review by the Taipei Medical University-Joint Institutional Review Board. The study was conducted according to all applicable guidelines and regulations set by the Health and Welfare Data Science Center. All analyses used de-identified, aggregated patient data.

### Statistical analysis

Descriptive statistics were used to characterise the demographic and clinical characteristics of the study cohort at the index date. Continuous data were expressed as mean and standard deviation (SD). The annual incidence of co-occurrence of thrombosis and thrombocytopenia diagnosis codes per 100 000 population was calculated by dividing the number cases each year by the size of the Taiwan population, estimated by the annual mid-year population for Taiwan [11], and stratified by age-group and sex. 95% confidence intervals (CI) were calculated using the exact method [12]. Kaplan-Meier analysis was used to estimate the survival probability after thrombosis with thrombocytopenia. The analysis was conducted using SAS Version 9.4 (Cary, NC, USA).

The funders of the study were involved in the study design and writing of the report.

## Results

**Fig 1:**
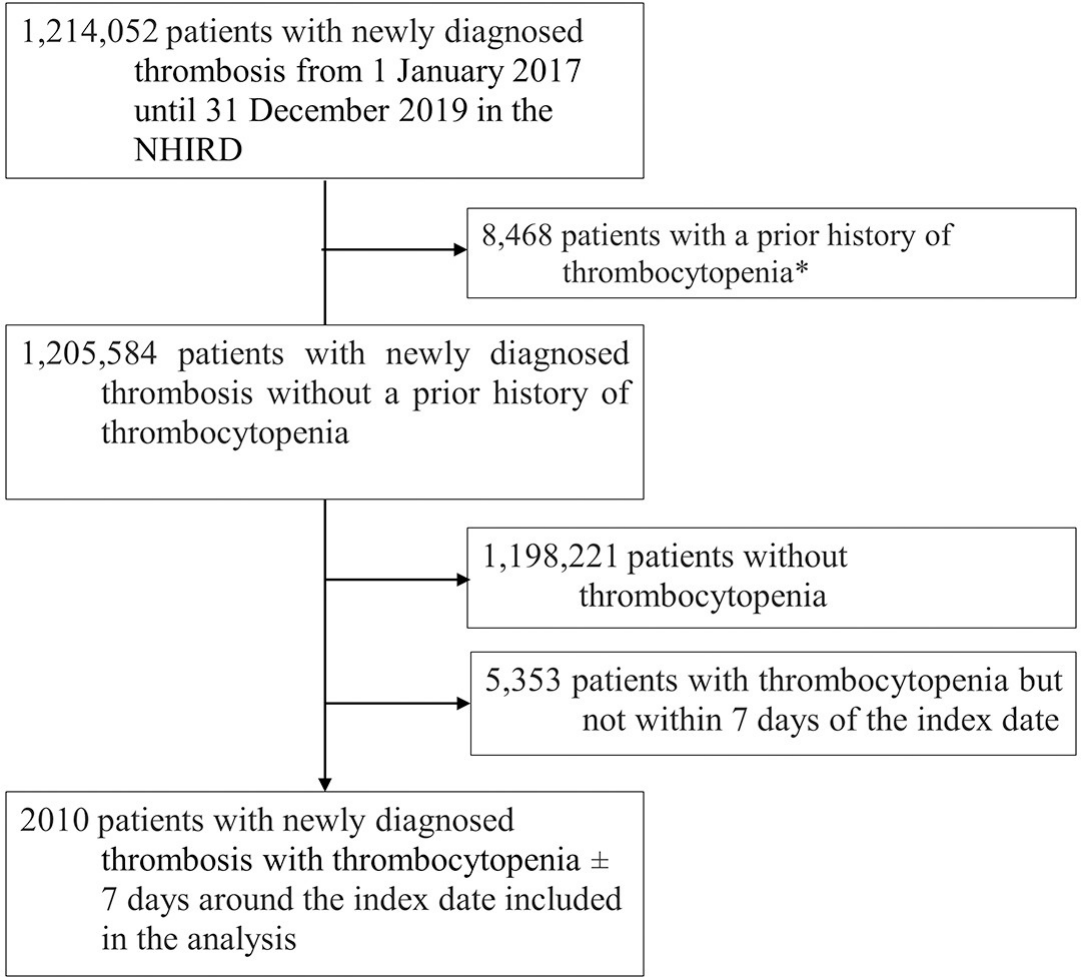
Flow diagram of patient enrolment NHIRD, National Health Insurance Research Database. *Patients with a diagnosis of thrombocytopenic up until the index date *minus* 7 days.

There were 7363 patients in the NHIRD with new co-occurring diagnoses of thrombosis and thrombocytopenia between 01 January 2017 and 31 December 2019 (Fig 1). Of these patients, we excluded 5353 patients in whom thrombocytopenia did not occur inside the 7-day window around the thrombosis index date, leaving 2010 patients in the analysis cohort. The mean age of patients over the 3-year study period was 64.71 years (SD 17.80) and the female to male ratio was 1: 1.45 (Table 1). Comorbid cardiovascular disease was common in the study population (58.66% of patients), as was comorbid hypertension (42.69%), diabetes (21.00%), solid tumour malignancy (16.22%), liver (13.38%), and renal (11.84%) impairment. Baseline demographic and clinical features were similar across the individual study years.

**Table 1:** Demographic and baseline characteristics of patients with co-occurring diagnosis codes for thrombosis and thrombocytopenia.

	Total	2017	2018	2019
	(n = 2,010)	(n = 837)	(n = 657)	(n = 516)
**Gender**				
Female	819 (40.81)	358 (42.82)	247 (37.71)	214 (41.47)
Male	1188 (59.19)	478 (57.18)	408 (62.29)	302 (58.53)
Missing	3	1	2	0
**Age (years)**				
**Mean (SD)**	64.71 (17.8)	64.91 (17.82)	64.47 (18)	64.68 (17.54)
0–9	24 (1.19)	9 (1.08)	7 (1.07)	8 (1.55)
10–19	16 (0.8)	6 (0.72)	5 (0.76)	5 (0.97)
20–29	33 (1.64)	14 (1.67)	14 (2.13)	5 (0.97)
30–39	94 (4.68)	44 (5.26)	31 (4.72)	19 (3.68)
40–49	193 (9.6)	81 (9.68)	61 (9.28)	51 (9.88)
50–59	334 (16.62)	139 (16.61)	112 (17.05)	83 (16.09)
60–69	482 (23.98)	186 (22.22)	162 (24.66)	134 (25.97)
70–79	380 (18.91)	160 (19.12)	116 (17.66)	104 (20.16)
80–89	343 (17.06)	157 (18.76)	109 (16.59)	77 (14.92)
≥ 90	111 (5.52)	41 (4.9)	40 (6.09)	30 (5.81)
**Clinical Characteristics**				
Cardiovascular diseases	1179 (58.66)	500 (59.74)	388 (59.06)	291 (56.4)
Hypertension	858 (42.69)	357 (42.65)	287 (43.68)	214 (41.47)
Diabetes	422 (21)	174 (20.79)	130 (19.79)	118 (22.87)
Liver impairment	269 (13.38)	125 (14.93)	87 (13.24)	57 (11.05)
Renal impairment	238 (11.84)	96 (11.47)	82 (12.48)	60 (11.63)
Haematological disease	9 (0.45)	^a^ (<0.5)	5 (0.76)	^a^ (<0.5)
Thrombophilia	4 (0.2)	^a^ (<0.5)	0 (0)	^a^ (<0.5)
Malignant neoplastic disease				
Solid tumour	326 (16.22)	125 (14.93)	111 (16.89)	90 (17.44)
Haematological malignancy	85 (4.23)	35 (4.18)	28 (4.26)	22 (4.26)
Pregnant	^a^ (<0.1)	^a^ (<0.1)	^a^ (<0.1)	^a^ (<0.1)

Data are mean (SD) or n (%).

^a^ Counts of ≤3 are hidden to protect patient privacy.

The incidence rate of co-occurring thrombosis and thrombocytopenia diagnoses was 3.55 per 100 000 population in 2017, 2.79 per 100 000 population in 2018, and 2.19 per 100 000 population in 2019, decreasing significantly over the study period (Cochran-Armitage test for trend ≤ 0.001). To explore this finding, we conducted a secondary analysis of the full population of 1 214 052 patients with newly diagnosed thrombosis in the NHIRD, of whom the vast majority (1 198 221, 98.7%) did not have associated thrombocytopenia. The incidence of any newly diagnosed thrombosis decreased in Taiwan over the study period, from 19.73 per 100 000 population in 2017, 16.74 per 100,000 in 2018, and 15.03 per 100 000 in 2019 (Cochran-Armitage test for trend ≤ 0.001).

The most frequent thrombotic events in patients with co-occurring thrombosis with thrombocytopenia diagnosis codes observed in the study population were events related to coronary artery disease (18.81%), cerebral infarction (16.87%), and disseminated intravascular coagulation (13.13%). The incidence of cerebral venous sinus thrombosis was low (<0.1%). The type and distribution of thrombotic events was similar over the study years (Table 2).

**Table 2:** Types of thrombosis in patients with co-occurring diagnosis codes for thrombosis and thrombocytopenia.

	Total	2017	2018	2019
	(n = 2,010)	(n = 837)	(n = 657)	(n = 516)
Coronary artery occlusion, thrombosis, embolism-including IHD and angina	378 (18.81)	164 (19.59)	107 (16.29)	107 (20.74)
Cerebral infarction	339 (16.87)	132 (15.77)	126 (19.18)	81 (15.7)
Disseminated intravascular coagulation	264 (13.13)	112 (13.38)	83 (12.63)	69 (13.37)
Myocardial Infarction and complication	183 (9.1)	74 (8.84)	58 (8.83)	51 (9.88)
Cerebral and Vertebral artery occlusion and embolism and transient ischaemic attack	135 (6.72)	54 (6.45)	49 (7.46)	32 (6.2)
Arterial thrombosis and embolism including extremities	87 (4.33)	41 (4.9)	22 (3.35)	24 (4.65)
Deep Vein Thrombosis (narrow)	59 (2.94)	20 (2.39)	20 (3.04)	19 (3.68)
Portal Vein thrombosis (include visceral, mesenteric)	43 (2.14)	12 (1.43)	20 (3.04)	11 (2.13)
Pulmonary Embolism	40 (1.99)	16 (1.91)	14 (2.13)	10 (1.94)
Intestinal infarction	25 (1.24)	8 (0.96)	12 (1.83)	5 (0.97)
Other venous embolism and thrombosis (including retinal)	19 (0.95)	8 (0.96)	7 (1.07)	4 (0.78)
Intraabdominal- splenic infarction and thrombosis	12 (0.6)	7 (0.84)	^a^ (<0.5)	^a^ (<0.5)
Cerebral venous sinus thrombosis (narrow)	^a^ (<0.1)	^a^ (<0.1)	^a^ (<0.1)	^a^ (<0.1)
Other Intraabdominal Venus thrombosis	0 (0)	0 (0)	0 (0)	0 (0)

Data are mean (SD) or n (%).

^a^ Counts of ≤3 are hidden to protect patient privacy.

**Fig 2:**
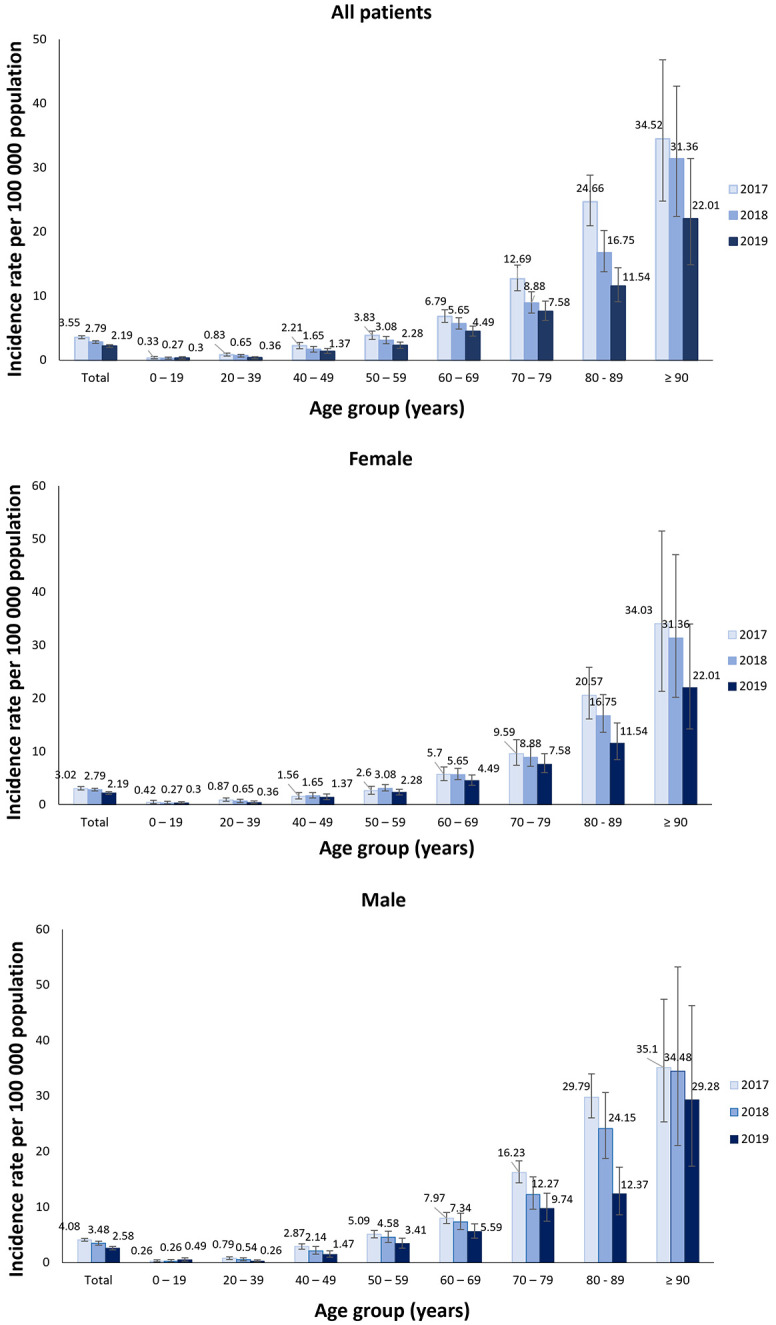
Annual incidence rate of co-occuring diagnosis codes for thrombosis and thrombocytopenia by age and sex. Tabulated data are provided in the supplementary material (S1 Table).

**Fig 3:**
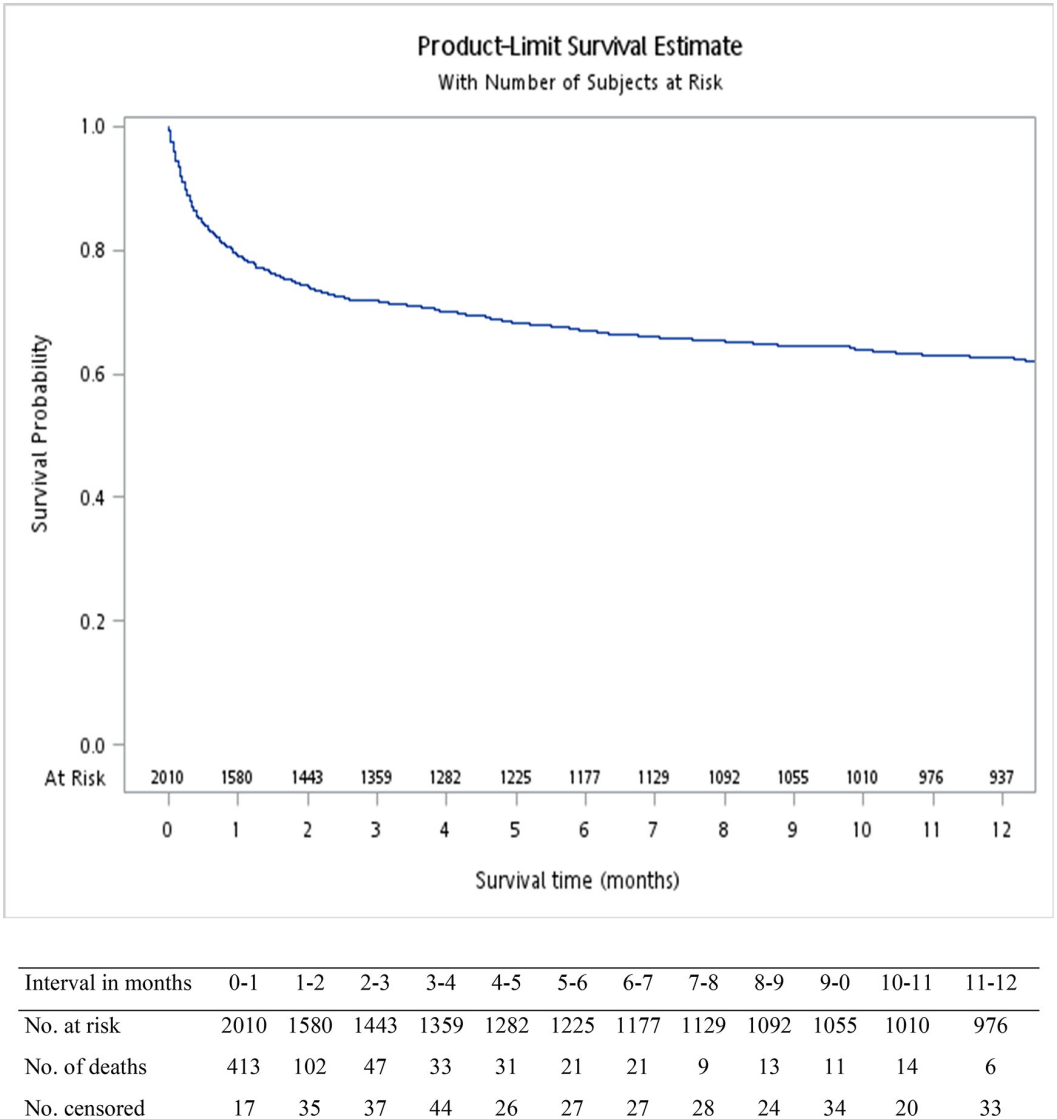
Survival curve of patients with new co-occuring diagnosis codes for thrombosis and thrombocytopenia.

The incidence rate of thrombosis with thrombocytopenia increased steadily with age; from an overall mean of 0.30 per 100 000 population (95% CI 0.22 to 0.41) in 0 to 19 year-olds, 0.62 per 100 000 population (95% CI 0.52 to 0.73) in 20 to 39 year-olds, and 29.0 per 100 000 population (95% CI 23.98 to 34.80) in adults aged 90 years and older (Fig 2). The overall observed incidence rate was higher in men (3.38 per 100 000 population, 95% CI 3.19 to 3.58) than women (2.30 per 100 000 population, 95% CI 2.15 to 2.46) except in the age-group of 20 to 39 year-olds where a higher incidence was observed in young females (0.69, 95% CI 0.54 to 0.87 *vs* 0.53, 95% CI 0.41 to 0.69). Overall, <0.1% of female patients were pregnant. 20.6% of patients died within the first month after the occurrence of thrombosis with thrombocytopenia (Fig 3).

## Discussion

We applied an algorithm to identify patients with co-occurring thrombosis and thrombocytopenia diagnosis codes in the NHIRD over a three-year period prior to the onset of the SARS-CoV-2 pandemic. The baseline incidence of the co-occurring diagnosis codes in the population of Taiwan was between 2.19 to 3.55 per 100 000 population between 2017 and 2019.

The demographic and clinical characteristics of patients with thrombosis with co-occurring thrombocytopenia were strikingly different from the characteristics of patients who have experienced TTS after COVID-19 vaccination [3,13]. Our study population was comprised of more men than women, more older persons than younger persons, and a high percentage of patients with comorbid disease, whereas cases of TTS tend to cluster in young and otherwise healthy persons, and more commonly in women after COVID-19 vaccination. Moreover, the majority of thrombotic episodes in our cohort were related to cerebrovascular and cardiac disease and cerebral venous sinus thrombosis was rare over the 3 study years, whereas cerebral venous sinus thrombosis is the most common presentation of TTS [3,13–15]. These differences likely represent a markedly different pathophysiology for TTS which is currently still being elucidated [16,17].

We observed a significant decrease in the incidence rate over the 3 years of the study that appeared to be due to a decrease in the overall rate of thrombosis in each study year (≤ 0.001). Improvements in the prophylactic management of thrombosis in patients at risk, including increased use of non-vitamin K agonist anticoagulants in Taiwan, may be responsible for this observation [18]. However, a longer study period is needed to confirm this trend because claims data from end of year medical episodes may be incomplete and finalised the year after.

Claims databases have been used to estimate baseline incidence rates of adverse events of interest that could arise after the introduction of new vaccines [19,20]. How these events are defined within the database has an important impact on the estimates obtained. Diseases defined by ICD codes alone potentially overestimate disease incidence due to false positive diagnoses resulting from coding errors or changes to the diagnosis once more data are obtained. ICD codes are therefore often combined with other parameters to reduce the risk of false positive diagnoses. Evidence of appropriate care/treatment can further increase the specificity of the case definition but can miss patients when the condition has no standard treatment. On the other hand, ICD-10 codes can lead incomplete capture of diagnoses if they are present in the medical record but not transcribed into an ICD code. A study of potential vaccine-associated adverse events in the United States found that incidence rates of adverse events were higher when more sensitive case definitions were employed compared to more specific definitions [19]. Conditions with a high degree (>90%) of concordance between sensitive and specific conditions included autoimmune hepatitis and cardiac conditions, while <20% concordance was observed for haemolytic anaemia, pancytopenia and acute disseminated encephalitis [19]. We elected to use a definition that did not include evidence of care/treatment in the algorithm because the most appropriate treatment for TTS was initially not known and continues to evolve [21].

Only one prior study using secondary, electronic healthcare data has evaluated pre-pandemic incidence rates of co-occurring thrombosis and thrombocytopenia diagnosis codes [22]. The study used data from seven databases in six European countries and recorded incidence rates for thromboses at specific anatomical sites of interest. The study confirmed the rarity of co-occurrence of thrombosis and thrombocytopenia diagnosis codes. There was heterogeneity in the estimated incidence rates across databases, ranging from 0.1 (splanchnic vein thrombosis with thrombocytopenia) to >400 (myocardial infarction or ischemic stroke with thrombocytopenia) cases per 100 000 person-years depending on the outcome studied and the database used. This heterogeneity is likely a result of differences in coding terms and/or availability of laboratory data for some, but not all databases. No data on mortality were reported, highlighting the utility of the NHIRD in terms of linkage to the death registry and estimation of survival after these events.

## Conclusions

Our study proposed a case definition to identify cases of co-occurring thrombosis and thrombocytopenia as a proxy for TTS in patients using the NHIRD. The rate of thrombosis with thrombocytopenia was low during the pre- COVID pandemic period, occurring most frequently in elderly males with multiple comorbidities. The cases obtained using this case definition provide a baseline contextual picture of the incidence and demographic features of individuals with thrombosis with co-occurring thrombocytopenia prior to the SARS-CoV-2 pandemic and the introduction of COVID-19 vaccines, which differ from the characteristics typically observed in cases of TTS. Therefore, further refinement of the case definition would be required in the post-COVID-19 vaccination era.

## Supporting information

S1 Table Sex-specific and age-specific incidence of thrombosis with thrombocytopenia in Taiwan from 2017 to 2019. (DOCX)
