# Supplementary material for: The incidence of thrombosis with co-occurring thrombocytopenia prior to the SARS-CoV2 pandemic: A population-based study
Source: PLoS One. 2024 May 24;19(5):e0301359. doi: 10.1371/journal.pone.0301359 (PMC11125481; doi:10.1371/journal.pone.0301359)
Supplement: S1 Table — (DOCX) [file pone.0301359.s001.docx]

**The incidence of thrombosis with co-occurring thrombocytopenia prior to the SARS-CoV2 pandemic: a population-based study Supplementary Material**

Yanfang Liu, Choo-Hua Goh, Dereck Shen, Hong Qiu_,_ Kuan-Chih Huang, Man Luo. Zhangjing Chen Chao-Hsiun Tang

# Table S1. Sex-specific and age-specific incidence of thrombosis with thrombocytopenia in Taiwan from 2017 to 2019

| **Age group** | **Total** | | | **Male** | | | **Female** | | |
| --- | --- | --- | --- | --- | --- | --- | --- | --- | --- |
|  | **Number of events** | **Person-years** | **Incidence rate per 100 000 person-years (95% CI)** | **Number of events** | **Person-years** | **Incidence rate per 100 000 person-years (95% CI)** | **Number of events** | **Person-years** | **Incidence rate per 100 000 person-years (95% CI)** |
| **2017** |  |  |  |  |  |  |  |  |  |
| Total | 837 | 23 555 522 | 3.55 (3.32–3.80) | 478 | 11 719 425 | 4.08 (3.72–4.46) | 358 | 11 836 097 | 3.02 (2.72–3.35) |
| 0–19 | 15 | 4 513 301 | 0.33 (0.19–0.55) | 6 | 2 350 106 | 0.26 (0.09–0.56) | 9 | 2 163 196 | 0.42 (0.19–0.79) |
| 20–39 | 58 | 6 988 827 | 0.83 (0.63–1.07) | 28 | 3 547 570 | 0.79 (0.52–1.14) | 30 | 3 441 257 | 0.87 (0.59–1.24) |
| 40–49 | 81 | 3 666 992 | 2.21 (1.75–2.75) | 52 | 1 808 930 | 2.87 (2.15–3.77) | 29 | 1 858 062 | 1.56 (1.05–2.24) |
| 50–59 | 139 | 3 631 404 | 3.83 (3.22–4.52) | 91 | 1 786 986 | 5.09 (4.10–6.25) | 48 | 1 844 418 | 2.60 (1.92–3.45) |
| 60–69 | 186 | 2 738 128 | 6.79 (5.85–7.84) | 105 | 1 316 925 | 7.97 (6.52–9.65) | 81 | 1 421 204 | 5.70 (4.53–7.08) |
| 70–79 | 160 | 1 261 325 | 12.69 (10.80–14.81) | 93 | 572 847 | 16.23 (13.10–19.89) | 66 | 688 478 | 9.59 (7.41–12.20) |
| 80 - 89 | 157 | 636 764 | 24.66 (20.95–28.83) | 84 | 281 938 | 29.79 (23.76–36.89) | 73 | 354 826 | 20.57 (16.13–25.87) |
| ≥ 90 | 41 | 118 782 | 34.52 (24.77–46.83) | 19 | 54 124 | 35.10 (21.14–54.82) | 22 | 64 658 | 34.03 (21.32–51.51) |
| **2018** |  |  |  |  |  |  |  |  |  |
| Total | 657 | 23 580 080 | 2.79 (2.58–3.01) | 408 | 11 716 247 | 3.48 (3.15–3.84) | 247 | 11 863 833 | 2.08 (1.83–2.36) |
| 0–19 | 12 | 4 408 219 | 0.27 (0.14–0.48) | 6 | 2 294 754 | 0.26 (0.10–0.57) | 6 | 2 113 465 | 0.28 (0.10–0.62) |
| 20–39 | 45 | 6 887 034 | 0.65 (0.48–0.87) | 19 | 3 502 043 | 0.54 (0.33–0.85) | 25 | 3 384 992 | 0.74 (0.48–1.09) |
| 40–49 | 61 | 3 694 766 | 1.65 (1.26–2.12) | 39 | 1 818 996 | 2.14 (1.52–2.93) | 21 | 1 875 770 | 1.12 (0.69–1.71) |
| 50–59 | 112 | 3 638 494 | 3.08 (2.53–3.70) | 82 | 1 789 423 | 4.58 (3.64–5.69) | 30 | 1 849 071 | 1.62 (1.09–2.32) |
| 60–69 | 162 | 2 866 169 | 5.65 (4.82–6.59) | 101 | 1 376 599 | 7.34 (5.98–8.92) | 61 | 1 489 570 | 4.10 (3.13–5.26) |
| 70–79 | 116 | 1 306 954 | 8.88 (7.33–10.65) | 73 | 594 860 | 12.27 (9.62–15.43) | 43 | 712 094 | 6.04 (4.37–8.13) |
| 80 - 89 | 109 | 650 914 | 16.75 (13.75–20.20) | 68 | 281 572 | 24.15 (18.75–30.62) | 41 | 369 343 | 11.10 (7.97–15.06) |
| ≥ 90 | 40 | 127 532 | 31.36 (22.41–42.71) | 20 | 58 002 | 34.48 (21.06–53.25) | 20 | 69 530 | 28.76 (17.57–44.42) |
| **2019** |  |  |  |  |  |  |  |  |  |
| Total | 516 | 23 596 027 | 2.19 (2.00–2.38) | 302 | 11 709 050 | 2.58 (2.30–2.89) | 214 | 11 886 977 | 1.80 (1.57–2.06) |
| 0–19 | 13 | 4 315 492 | 0.30 (0.16–0.52) | 11 | 2 245 745 | 0.49 (0.24–0.88) | 2 | 2 069 748 | 0.10 (0.01–0.35) |
| 20–39 | 24 | 6 749 988 | 0.36 (0.23–0.53) | 9 | 3 438 536 | 0.26 (0.12–0.50) | 15 | 3 311 452 | 0.45 (0.25–0.75) |
| 40–49 | 51 | 3 729 457 | 1.37 (1.02–1.80) | 27 | 1 833 732 | 1.47 (0.97–2.14) | 24 | 1 895 726 | 1.27 (0.81–1.88) |
| 50–59 | 83 | 3 642 108 | 2.28 (1.82–2.83) | 61 | 1 789 353 | 3.41 (2.61–4.38) | 22 | 1 852 755 | 1.19 (0.74–1.80) |
| 60–69 | 134 | 2 982 706 | 4.49 (3.76–5.32) | 80 | 1 431 011 | 5.59 (4.43–6.96) | 54 | 1 551 696 | 3.48 (2.61–4.54) |
| 70–79 | 104 | 1 372 420 | 7.58 (6.19–9.18) | 61 | 626 183 | 9.74 (7.45–12.51) | 43 | 746 237 | 5.76 (4.17–7.76) |
| 80 - 89 | 77 | 667 534 | 11.54 (9.10–14.42) | 35 | 283 024 | 12.37 (8.61–17.20) | 42 | 384 510 | 10.92 (7.87–14.76) |
| ≥ 90 | 30 | 136 323 | 22.01 (14.85–31.42) | 18 | 61 468 | 29.28 (17.36–46.28) | 12 | 74 855 | 16.03 (8.28–28.00) |
| **2017-2019** |  |  |  |  |  |  |  |  |  |
| Total | 2010 | 70 731 629 | 2.84 (2.72–2.97) | 1188 | 35 144 722 | 3.38 (3.19–3.58) | 819 | 35 586 907 | 2.30 (2.15–2.46) |
| 0–19 | 40 | 13 237 012 | 0.30 (0.22–0.41) | 23 | 6 890 605 | 0.33 (0.22–0.49) | 17 | 6 346 409 | 0.27 (0.16–0.42) |
| 20–39 | 127 | 20 625 849 | 0.62 (0.52–0.73) | 56 | 10 488 149 | 0.53 (0.41–0.69) | 70 | 10 137 701 | 0.69 (0.54–0.87) |
| 40–49 | 193 | 11 091 215 | 1.74 (1.51–2) | 118 | 5 461 658 | 2.16 (1.80–2.58) | 74 | 5 629 558 | 1.31 (1.04–1.64) |
| 50–59 | 334 | 10 912 006 | 3.06 (2.75–3.4) | 234 | 5 365 762 | 4.36 (3.83–4.95) | 100 | 5 546 244 | 1.80 (1.48–2.18) |
| 60–69 | 482 | 8 587 003 | 5.61 (5.13–6.13) | 286 | 4 124 535 | 6.93 (6.17–7.77) | 196 | 4 462 470 | 4.39 (3.81–5.04) |
| 70–79 | 380 | 3 940 699 | 9.64 (8.71–10.65) | 227 | 1 793 890 | 12.65 (11.09–14.38) | 152 | 2 146 809 | 7.08 (6.02–8.28) |
| 80–89 | 343 | 1 955 212 | 17.54 (15.76–19.47) | 187 | 846 534 | 22.09 (19.09–25.43) | 156 | 1 108 679 | 14.07 (11.99–16.41) |
| ≥ 90 | 111 | 382 637 | 29 (23.98–34.8) | 57 | 173 594 | 32.83 (25.10–42.24) | 54 | 209 043 | 25.83 (19.60–33.45) |

CI, confidence interval
